# Clinical impact of the methylation status of SMAD4 and AKR1B1 genes in a liquid biopsy sample as a prognostic marker for breast cancer

**DOI:** 10.1038/s41598-026-37937-6

**Published:** 2026-02-27

**Authors:** Menha Swellam, Amal Ramadan, Mohamed Emam Sobeih, Noha M. Bakr

**Affiliations:** 1 Biochemistry Department, Biotechnology Research Institute, National Research Centre, El-Bohouth Street, Dokki, Giza Egypt; 2 High Throughput Molecular and Genetic laboratory, Center of Excellence for Advanced Sciences, National Research Centre, El-Bohouth Street, Dokki, Giza Egypt; 3 Medical Oncology Department, National Cancer Institute, Cairo, Egypt

**Keywords:** Breast cancer, DNA methylation, Diagnosis, Prognosis, SMAD4 and AKR1B1, Biomarkers, Cancer, Oncology

## Abstract

The role of DNA methylation in the prognosis of breast cancer, particularly concerning small mothers against decapentaplegic 4 (SMAD4) and aldo-keto reductase family 1 member B1 (AKR1B1), remains largely unexplored. This study aimed to investigate the clinical role of SMAD4 and AKR1B1 methylation as noninvasive prognostic biomarkers in breast cancer. The study included 140 individuals. The patients were stratified into two groups based on their diagnostic investigation: women diagnosed with cancer in breast (*N* = 80) and cases with benign lesions in breast (*N* = 30). Additionally, a group of subjects considered as healthy served as the control group (*N* = 30). Methylation levels of SMAD4 and AKR1B1 were quantified using the Methyl II quantitative PCR system. The methylation specificity and sensitivity were examined through performing a receiver operating characteristic (ROC) curve analysis. The association of the methylation of investigated patterns with breast cancer clinical features and response to treatment was also examined. Survival patterns were assesed using Kaplan-Meier analyses. The outcomes revealed that hypermethylation of SMAD4 and AKR1B1 was upregulated in cancerous patients relative to the benign group and healthy subjects. Based on the values of the area under the curve (AUC) (0.945 and 0.935, respectively) for SMAD4 and AKR1B1, both markers demonstrated superior diagnostic accuracy, surpassing conventional biomarkers for instance cancer antigen 15 − 3 (CA 15 − 3; AUC = 0.698) as well as carcinoembryonic antigen (CEA; AUC = 0.537). Remarkably, SMAD4 and AKR1B1 hypermethylation exhibited a significant association with invasive duct carcinoma (IDC), particularly in early stages, high grades, and cases with lymph node metastasis. A significant difference was observed in methylation status concerning both items and treatment response. Additionally, survival outcomes indicated that hypermethylation of SMAD4 and AKR1B1 was associated with worse DFS and OS. In conclusion, SMAD4 and AKR1B1 methylation may serve as significant epigenetic markers affecting breast cancer prognosis, potentially indicating more aggressive disease and poorer outcomes in these patients.

## Introduction

 Breast cancer (BC) is categorized as the fifth major cause of tumor-associated mortality and represents a significant threat to the quality of life and overall health of numerous women worldwide^1,2^. Its etiology is multifactorial, involving a complex interplay of hormonal, environmental, genetic, and epigenetic factors^3^. Numerous studies indicate that epigenetic alterations, which are inherited alterations in the function and expression of genes that do not include the DNA sequence changes, are a primary factor in breast cancer^4,5^. During carcinogenesis, various epigenetic alterations including DNA methylation occur. Both aberrant DNA hypermethylation and hypomethylation have been shown to promote tumor suppressor genes stopping and oncogenes stimulation, respectively, in various tumor types, including breast cancer^6–9^.

The small mothers against decapentaplegic 4 (SMAD4) gene, located on human chromosome 18q21.1, was first identified in pancreatic carcinoma^10^, and encodes an intracellular protein integral to the transforming growth factor (TGF)-β signaling pathway which regulates nuclear transcription and tumor suppressor gene activation by mediating signal transduction from the surface of the cell to the nucleus^11^. In BREAST CANCER, SMAD4 inactivation is strongly associated with carcinogenesis and therapeutic resistance^12^. Dysregulation of SMAD family gene expression can disrupt signaling pathways, resulting in enhanced proliferation and compromised apoptosis of breast cancer cells. Furthermore, SMAD modulates TGF-β-induced epithelial-mesenchymal transition (EMT), and its inactivation contributes to the metastasis and invasion of breast cancer^13^. Drugs that target the TGF-β/SMAD signalling system may be therapeutically helpful for particular breast cancer^14^. SMAD4 gene expression levels in breast cancer tissues can be beneficial as a valuable prognostic biomarker and aid in predicting response to treatments^15^. As a result, the inactivation of SMAD4 will cause overgrowth making the tissue to develop cancerous disease^10,16^.

Aldo-keto reductase family 1 (AKR1) is one of the superfamily of aldose keto reductase (AKR), specifically AKR1B1which is situated on human chromosome 7q33 and encodes a transcript with 10 exons^17^. It is a catalyst that stimulates the glucose reduction to sorbitol and is involved in the osmoregulation and polyol pathway. It serves as a synthase for prostaglandin PGF2, and indirectly affects the protein kinase C pathway, thereby promoting inflammation and cell proliferation via the activation of nuclear factor kappa B^18,20^. AKR1B1 is linked to the progression of basal-like breast cancer, characterized by high-grade tumors and increased metastasis. AKR1B1 overexpression activates pathways that promote cancer stem cell traits, thereby increasing tumor aggressiveness^19^. The involvement of AKR1B1 in cancer remains incompletely elucidated. however, emerging evidence indicates its significant impact on cancer progression^20^.

The early diagnosis and prognosis of breast cancer pose significant challenges, and the role of methylation of promoter gene in the prognosis of BC remains largely ambiguous. The present study investigated the impact of gene methylation on breast cancer by quantitatively assessing the methylation levels of the SMAD4 and AKR1B1 genes in blood samples from breast cancer women, benign lesions, and control groups. It aimed to compare the diagnostic efficacy of these methylation patterns with traditional tumor markers, as well as to investigate their prognostic significance and implications for survival outcomes. The findings may enhance understanding of the breast carcinogenesis process guide clinical follow-up strategies, and inform targeted therapeutic approaches to mitigate breast cancer burden. According to available information, this represents the first study to elucidate the prognostic role of SMAD4 and AKR1B1 methylation among Egyptian women with breast cancer.

## Materials and methods

### Enrolled participants

This Egyptian study involved 120 participants categorized into three groups: Group I comprised 80 primary breast cancer cases, which were further divided into 47 patients with invasive ductal carcinoma (IDC), and 33 ductal carcinoma in situ (DCIS) cases. Group II included 30 cases of benign breast lesions. Group III comprised 10 participants who appeared to be healthy. Participants, including patients and healthy controls, were recruited from the Department of Medical Oncology at the National Cancer Institute in Cairo, Egypt. Comprehensive demographic and clinical-pathological data were collected for the recruited cancerous and benign patients. Based on established standard criteria, histological staging and grading for breast cancer patients were identified^21,22^. Hormone receptors were analyzed through an immunohistochemical technique as documented in prior studies^23^. Both estrogen and progesterone receptors were deemed positive through staining of ≥ 10% of nuclei in ten high-power fields, and the positive result for human epidermal growth factor receptor 2 (HER-2) neu was confirmed when recorded as + 3^24^.

Cases with a history of prior cancer and other malignancies were excluded. The research gained ethical approval (Approval number: 09241123) from the Committee for Medical Research Ethics at the National Research Centre located in Dokki, Giza, Egypt, adhering to the ethical guidelines for human research as delineated in the Declaration of Helsinki. All enrolled subjects gave their informed consent before participating in the study.

### Treatment strategies and patient follow-up

The majority of our patients diagnosed with invasive carcinoma presented with early or locally advanced stage and the main treatment strategy was surgery followed by adjuvant systemic therapy in the form of anthracycline and taxenes based therapy then postoperative radiotherapy based on many clinical, histological and surgical factors and the hormonal therapy based on menopausal status after that the patients were kept under follow up on regular basis. Patients who have HER2 + ve expression had received anti HER 2 therapy (trustuzumab) for about one year in the early stages. The molecular pattern of our patients was a prognostic and predictive factor as most our patients were luminal subtype. Few patients of our study population were diagnosed on advanced stage and were treated with palliative therapy depending on their molecular pattern. Regarding the patients who diagnosed as DCIS was treated by surgery followed by hormonal therapy in case of ER + ve disease they kept under follow up on regular basis. Regarding the non-malignant lesions were treated either by surgery only or follow up on regular basis.

As defined by the guidelines of Response Evaluation Criteria in Solid Tumors (RECIST) version 1.1, treatment response to neoadjuvant and adjuvant systemic therapies were evaluated primarily using physical examination and standard radiological imaging (e.g., ultrasound, mammography, and/or computed tomography scans), and classified into: complete response (CR) if there was a complete disappearance of all targeted lesions, patients with partial response (PR) when the sum of diameters of targeted lesions declined at least 30%, patients with stable disease (SD) when neither adequate reduction to qualify for PR nor enough elevate to qualify for Progressive Disease (PD), and patients with progressive disease (PD) when there was increase in the sum of diameters of targetes lesions at least a 20%, or the development of one or more new lesions. Time points for response were assessed at the completion of neoadjuvant therapy (if applicable) and regularly every 6 months for the first 5 years during active surveillance and follow-up. Survival analysis was progression-free survival (PFS), which represented the beginning of the initial systemic therapy strategy till the progression of the disease (local, regional, or distal recurrence), contralateral breast cancer, the manifestation of a second primary tumor, or death from any reason, and overall survival (OS) which estimated based on the first diagnosis of BC to the time of the final follow-up or death from any reson.

## Methods

###  Collection and processing of specimen

Five milliliters of whole blood were collected from the participants, then divided into two tubes: one for the evaluation of traditional tumor-related markers, while the other 2.5 ml was collected in a tube that included EDTA for genetic assessment.

### Measurement of tumor markers

Enzyme-linked immunosorbent assay (ELISA) kits (Immunospec Corporation, the Netherlands) were purchased to quantify levels of both serum carcinoembryonic antigen (CEA) and cancer antigen 15.3 (CA15-3) in accordance with the manufacturer’s protocol, and absorbance readings were measured through utilizing the GloMax^®^-Multi detection system (Promega, USA).

### Genomic DNA purification

DNA was extracted from whole blood samples according to the spin column approach by utilizing QIAamp DNA blood Min kit (Cat No: 51104, Qiagen, Germany), as defined by the manufacturer’s protocol recommendations. The extracted DNA template concentration and purity were determined via using a Nano-drop spectrophotometer (Quawell, Q-500, USA) and subsequently stored at − 80 °C until another molecular analysis.

### Methylation assessement

By using EpiTect Methyl II PCR System (Qiagen, Germany), the DNA methylation pattern was identified. This assay relies on the principle of identification of residual input genome following digestion using a restriction enzyme sensitive to methylation. This assay needs two phases: phase I: restriction digestion of DNA templates, which was done in accordance with the manufacturer’s instructions of the commercial (EpiTect Methyl II DNA Restriction kit, cat. no: 335452).

The DNA template was divided into 4 tubes: mock (M0), methylation-sensitive (Ms), M-dependent (Md), as well as M-sensitive-dependent enzyme (Msd). Subsequently, reaction mixtures were incubated for 6 h at 37 °C in a SureCycler 8800 thermal cycler (Agilent, California, USA). Once incubation was finished, the reactions were terminated by stopping the enzymes’ function at 65 °C for 20 min, followed by subsequent storage of the enzyme reactions at − 20 °C before quantitative PCR (qPCR). Then phase II: The methylation status of the examined genes was assessed using a quantitative polymerase chain reaction (qPCR) approach with the QPCR instrument (Stratagene, Agilent Technologies, CA). The enzyme reactions were added to RT2 qPCR SYBR Green/ROX Master Mix (Cat number: 330520), and were subsequently transferred into a PCR plate pre-filled with primer mixes (http://qiagen.com/us/shop/epigenetics/epitect-methyl-ii-pcr/assays/) (EpiTect Methyl II qPCR Primer Assay, Cat no. 335002)., specifically SMAD4 (Cat number EPHS104636-1 A [CpG Island 104636]), and AKR1B1 (Cat number EPHS113490-1 A [CpG Island 113490]). The PCR protocol began with a single cycle for 10 min at 95 °C, then three cycles for 30 s at 99 °C, and for 1 min at 72 °C. In conclusion, 40 cycles were conducted for 15 s at 97 °C and for 1 min at 72 °C. In the final step, the raw values of ^Δ^CT were inserted into the records analysis worksheet (EpiTect Methyl II PCR Array Microsoft Excel based data analysis template), which routinely analyzes the relative proportions of methylated and unmethylated DNA fractions.

### Impact of SMAD4 and AKR1B1 on survival status

Follow-up of BC patients was for a duration of 39 months, with a mean period of 31 months. Methylation patterns for SMAD4 and AKR1B1 were assessed concerning PFS and OS.

### Statistical analysis

The SPSS package software (version 16.0) was used to perform the statistical analysis of the input data. Data was statistically significant when the P-value (two-tailed) was recorded as < 0.05. Receiver operating characteristic (ROC) curve analysis was achieved to find the ideal cutoff point that achieves the highest combined sensitivity and specificity for discriminating between cancerous, benign, and control group^25^. ANOVA and chi-square tests were performed, as appropriate, to compare continuous and categorical variables across groups. Correlations between studied biomarkers were assessed using correlation coefficient (Spearman’s rank). Survival outcomes was done by the Kaplan-Meier analysis.

## Results

### Demographic and clinical features of the investigated individuals

Participants (*N* = 120) exhibited no significant age differences (F = 0.265, *P* value = 0.767). The study examined classical tumor markers specific to breast cancer (CEA and CA15.3) and the methylation patterns of SMAD4 and AKR1B1 across three groups. In a study involving breast cancer patients (group I, *N* = 80), participants were categorized into 69 pre-menopausal women and the remainder as post-menopausal women. Pathological types reported 33 cases of duct carcinoma in situ (DCIS) and 47 cases of invasive ductal carcinoma (IDC). According to clinical staging, 35 breast cancer patients were classified as early stage, while the remaining patients were categorized as late stage. Based on histological grade, the cases were categorized into low histological grading (*N* = 31) and high-grade breast tumors (*N* = 49). Thirty-four patients exhibited negative lymph node status and negative hormonal status; ER and PR were reported in 41 and 39 patients, respectively. Her-2/neu expression was observed in 28 patients diagnosed with breast cancer. Group II consisted of 30 patients diagnosed with benign breast lesions, including 11 with intraductal papillomatosis, 10 with follicular hyperplasia, and 9 with fibroadenoma. Group III was clinically enrolled as healthy individuals and designated as the control group. Demographic and clinicopathological criteria were summarized in Table 1.

### Associations of traditional tumor markers and investigated genes’ methylation patterns with breast cancer development among different investigated groups

**Fig. 1:**
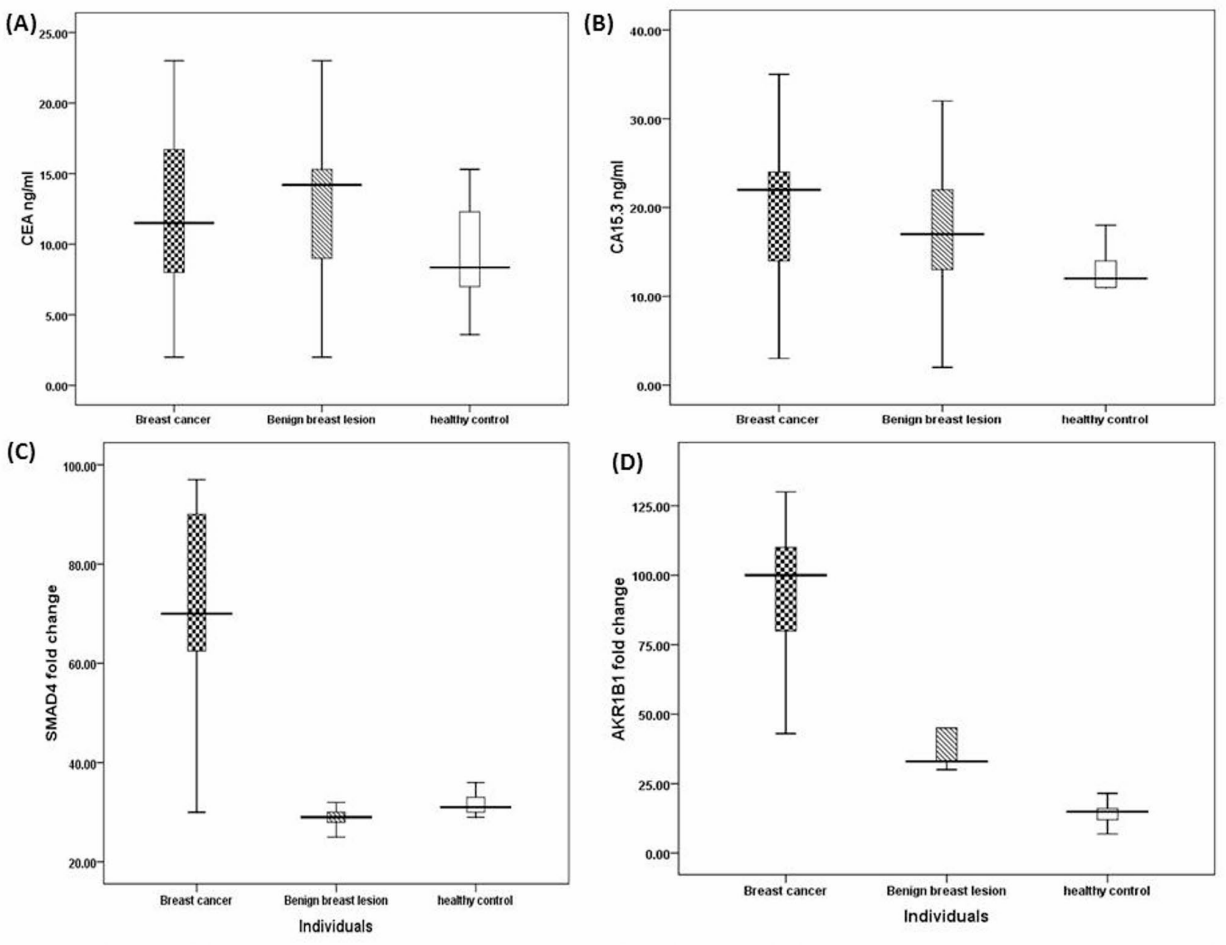
Box-plot comparing levels across groups. **A** level of CEA, **B** level of CA15.3, **C** level of SMAD4 and **D** level of AKR1B1 among investigated groups.

As shown in Table 2, traditional tumor markers of breast cancer including CEA and CA15.3, were elevated in breast cancer patients compared to benign breast lesion and healthy controls as expressed by mean ± SD (12 ± 0.7, 13.6 ± 1, and 9 ± 0.7, respectively for CEA (*P* = 0.01), and 19.5 ± 1, 16.7 ± 1.3, and 12 ± 1, respectively, for CA 15.3 (*P* < 0.001). Nevertheless, the ASMAD4 and AKR1B1 methylation patterns were noticeably (*P* < 0.0001) associated with breast cancer patients. As expressed by mean ± SD, breast cancer cases exhibited a significantly higher degree of SMAD4 methylation (72 ± 2) compared to benign lesions (34 ± 3) and healthy females (31 ± 0.5). Similarly, for the AKR1B1 methylation pattern, breast cancer cases revealed a significantly higher degree of methylation (94.4 ± 2.3) compared to benign lesions (50 ± 5) and healthy females (13 ± 0.8) as shown in Figure (1 a-d). Considering positivity rates, the cutoff points (> 15, > 30, >36, and > 45, for CEA, CA15.3, SMAD4, and AKR1B1, respectively), indicated that positivity rates within breast cancer cases, benign beast lesions, and healthy controls groups (27%, 20, and 0%, respectively) for CEA, CA15.3 (45%, 30, and 0%, respectively), as well as methylation status for SMAD4 (95%, 10, and 0%, respectively), and AKR1B1 (97.5%, 50%, and 0%, respectively) were significantly elevated in BC cases versus the other two groups.

**Table 1:** Demographic and clinic-pathological data for enrolled individuals.

Factors	Control *N* = 30	Benign breast lesions *N* = 30	Breast cancer *N* = 80
Age			
Median	47	45	50
Mean ± SD	50.7 ± 8	50 ± 8	51.4 ± 8
Menopausal status			
Pre-menopause	14	15	40
Post-menopause	16	15	40
Pathology status	---	Fibroadenoma (*n* = 9)	DCIS (*n* = 33) IDC (*n* = 47)
		Follicular hyperplasia (*n* = 10)	
		Intra-ductal papillomatosis (11)	
Clinical stage	---	---	
Early stage			35
Late stage			45
Histological grade	---	---	
Low grade			31
High grade			49
Lymph- node involvement	---	---	34
Negative			
Positive			
ER-status	---	---	
Negative			41
Positive			39
PgR-status	---	---	
Negative			39
Positive			41
HER-2neu-status	---	---	
Negative			52
Positive			28

**Table 2:** Mean (median) and methylation patterns of tumor markers and investigated genes among investigated groups.

Items	CEA (ng/ml)		CA15.3 (ng/ml)		SMAD4 (fold change)		AKR1B1 (fold change)	
	Mean	> 15 (*n*,%)	Mean	> 30 (*n*,%)	Mean	> 36 (*n*,%)	Mean	> 45(*n*,%)
Breast cancer	12 ± 0.7 (11)	22 (27%)	19.5 ± 1 (22)	36 (45%)	72 ± 2 (70)	75 (95%)	94.4 ± 2.3 (100)	78 (97.5%)
Benign	13.6 ± 1 (14)	6 (20%)	16.7 ± 1.3 (17)	9 (30%)	34 ± 3 (29)	3 (10%)	50 ± 5 (33)	15 (50%)
Control	9 ± 0.7 (8.4)	0 (0%)	12 ± 1 (12)	0 (0%)	31 ± 0.5 (31)	0 (0%)	13 ± 0.8 (15)	0 (0%)
Statistics Between three groups	F= 4.7, *P* = 0.01	*X*^*2*^= 10, *P* = 0.006	F = 8.5, *P*<0.001	*X*^*2*^= 20, *P*<0.001	F = 116, *P*<0.001	*X*^*2*^= 113, *P*<0.001	F = 187, *P*<0.001	*X*^*2*^= 51, *P*<0.001
Cancer vs. Control	F= 5.2, *P* = 0.024	*X*^*2*^= 10, *P* = 0.001	F= 15, *P*<0.0001	*X*^*2*^= 20, *P*<0.0001	F= 176, *P*<0.0001	*X*^*2*^= 92, *P*<0.0001	F= 480, *P*<0.0001	*X*^*2*^= 100, *P*<0.0001
Cancer vs. Benign	F= 1.4, *P* = 0.232	*X*^*2*^= 0.647, *P* = 0.471	F= 2.2, *P* = 0.139	*X*^*2*^= 2, *P* = 0.154	F= 106, *P*<0.0001	*X*^*2*^= 77, *P*<0.0001	F= 83, *P*<0.0001	*X*^*2*^= 37, *P*<0.0001
Benign vs. Control	F= 14, *P*<0.0001	*X*^*2*^= 6.7, *P* = 0.01	F= 7.2, *P* = 0.009	*X*^*2*^= 10.6, *P* = 0.001	F= 1.2, *P* = 0.282	*X*^*2*^= 3.1, *P* = 0.07	F= 44, *P*<0.0001	*X*^*2*^= 20, *P*<0.0001

###  The diagnostic efficacy of SMAD4 and AKR1B1 methylation status

**Fig. 2:**
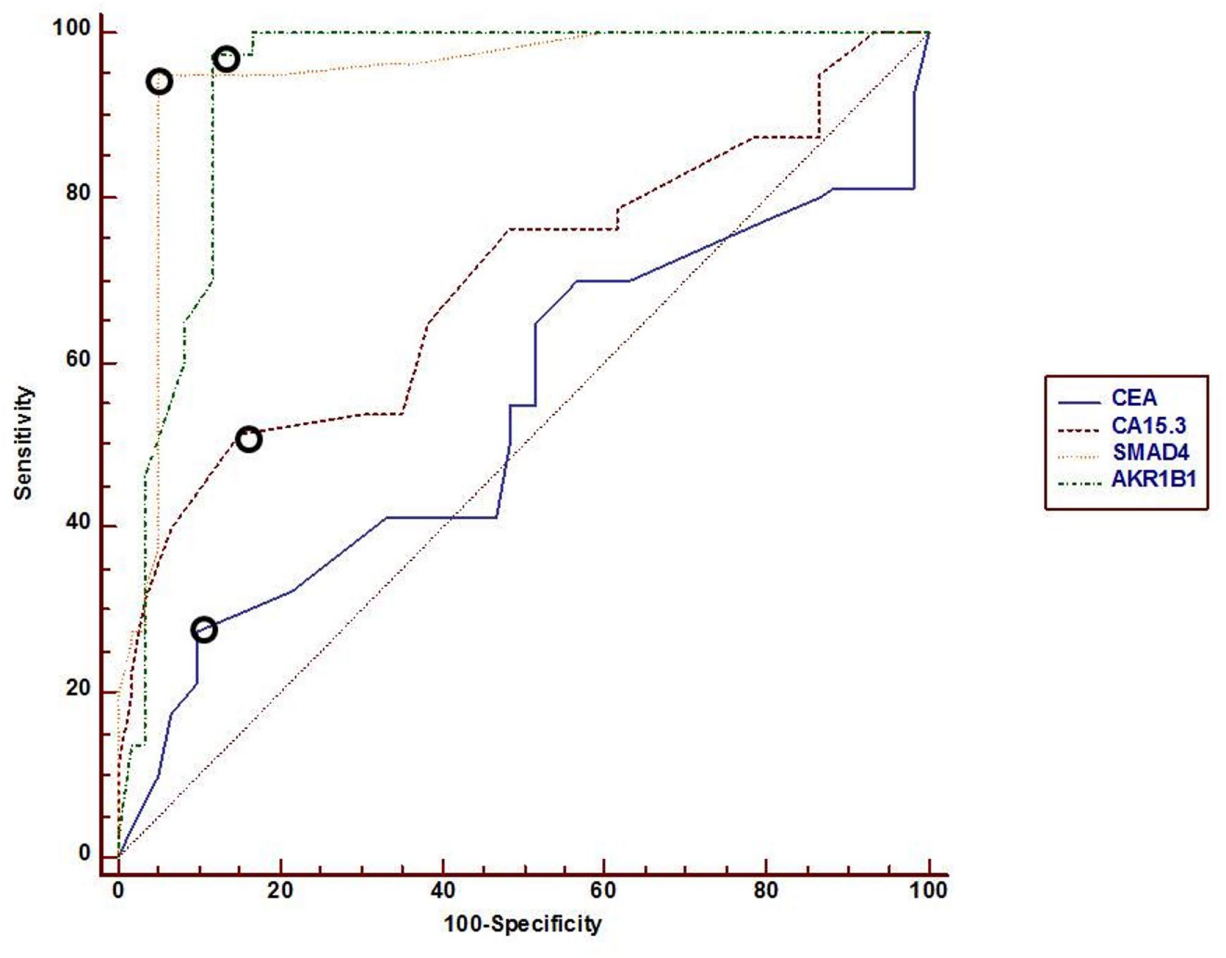
Receiver operating characteristic (ROC) curve for detection of diagnostic efficacy among investigated markers. Open circles donate the best cutoff points.

To investigate its effectiveness in diagnosis, analysis of ROC curve was plotted for SMAD4 and AKR1B1 methylation, with the ideal cutoff point was identified (Fig. 2) which discriminates between cancer and non-cancer patients. As presented in Table 3, The highest AUC [95% CI] was observed for SMAD4 (0.945), followed by AKR1B1 (0.935), then CA15.3 (0.698), and CEA (0.537) exhibited the lowest AUC. Moreover, the sensitivity and specificity of SMAD4 (95%, and 95%, respectively) exceed those of CEA (27.50%, and 90%, respectively) and CA15.3 (51.25%, and 85%, respectively), whereas AKR1B1 demonstrates superior sensitivity (97.50%) compared to CEA and CA15.3.

**Table 3:** Cutoff points, AUC, sensitivity and specificity for investigated items.

Items	Cutoff point	95% CI	AUC	Sensitivity	95% CI	Specificity	95% CI
CEA	15 ng/ml	0.451–0.621	0.537	27.50	18.1–38.6	90.00	79.5–96.2
CA15.3	30 ng/ml	0.615–0.772	0.698	51.25	39.8–62.6	85.00	73.4–92.9
***SMAD4***	36 fold change	0.894–0.977	0.945	95.00	87.7–98.6	95.00	86.1–98.9
***AKR1B1***	45 fold change	0.880–0.969	0.935	97.50	91.2–99.6	88.33	77.4–95.2

### Levels of examined parameters and clinicopathological data

High methylation levels of SMAD4 and AKR1B1 were observed in IDC (76.6 ± 2.6 and 100 ± 2.7) compared to DCIS (66.6 ± 2.5 and 89.5 ± 3.6, respectively) at (F = 6.5, *P* = 0.012 and F = 6.3, *P* = 0.015, respectively). Furthermore, methylation levels increased significantly in late-stage cases (76.6 ± 2.7 and 99.7 ± 2.8) compared to early stage cases (67 ± 2.4 and 91.4 ± 3.6, respectively) at (F = 6, *P* = 0.017 and F = 4.3, *P* = 0.04, respectively). Similarly, methylation patterns for SMAD4 and AKR1B1 were increased significantly with high-grade breast cancer (76.6 ± 2.5 and 100 ± 2.6) as compared to low-grade (65 ± 2.4 and 88 ± 3.2, respectively) at (F = 7.8, *P* = 0.006 and F = 6.4, *P* = 0.014, respectively). Methylation patterns for SMAD4 reported a significant difference (F = 4.2, *P* = 0.043) with lymph node (LN) status, as methylation increases with positive LN (68 ± 2.8) as compared to negative LN (7.6 ± 2.9). No significant difference was reported between CEA, CA15.3, and clinical pathological parameters.

### Correlation between methylation patterns and tumor markers

**Fig. 3:**
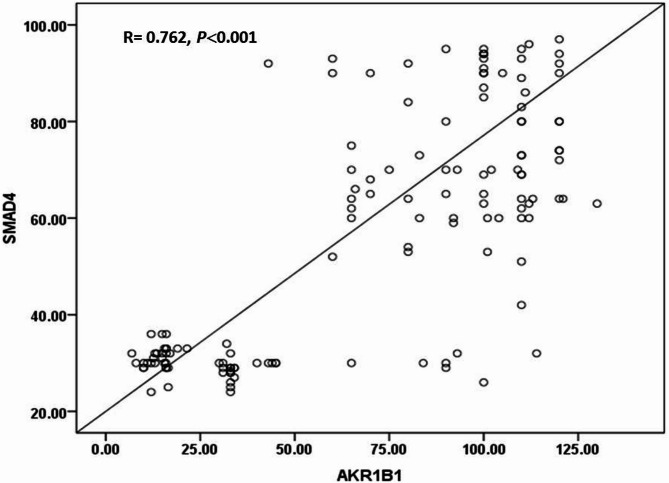
Correlation between methylation patterns. Significant difference was observed between SMAD4 and AKR1B1 methylation patterns.

No significant association was observed between the methylation patterns of the enrolled individuals and tumor markers (*n* = 120). On the other hand, a significant difference was observed between SMAD4 and AKR1B1 among breast cancer, as both reported an increased levels in breast cancer patients as demonstrated in Fig. 3.

### Efficacy of the examined parameters in identifying early-stage and low-grade tumors

**Fig. 4:**
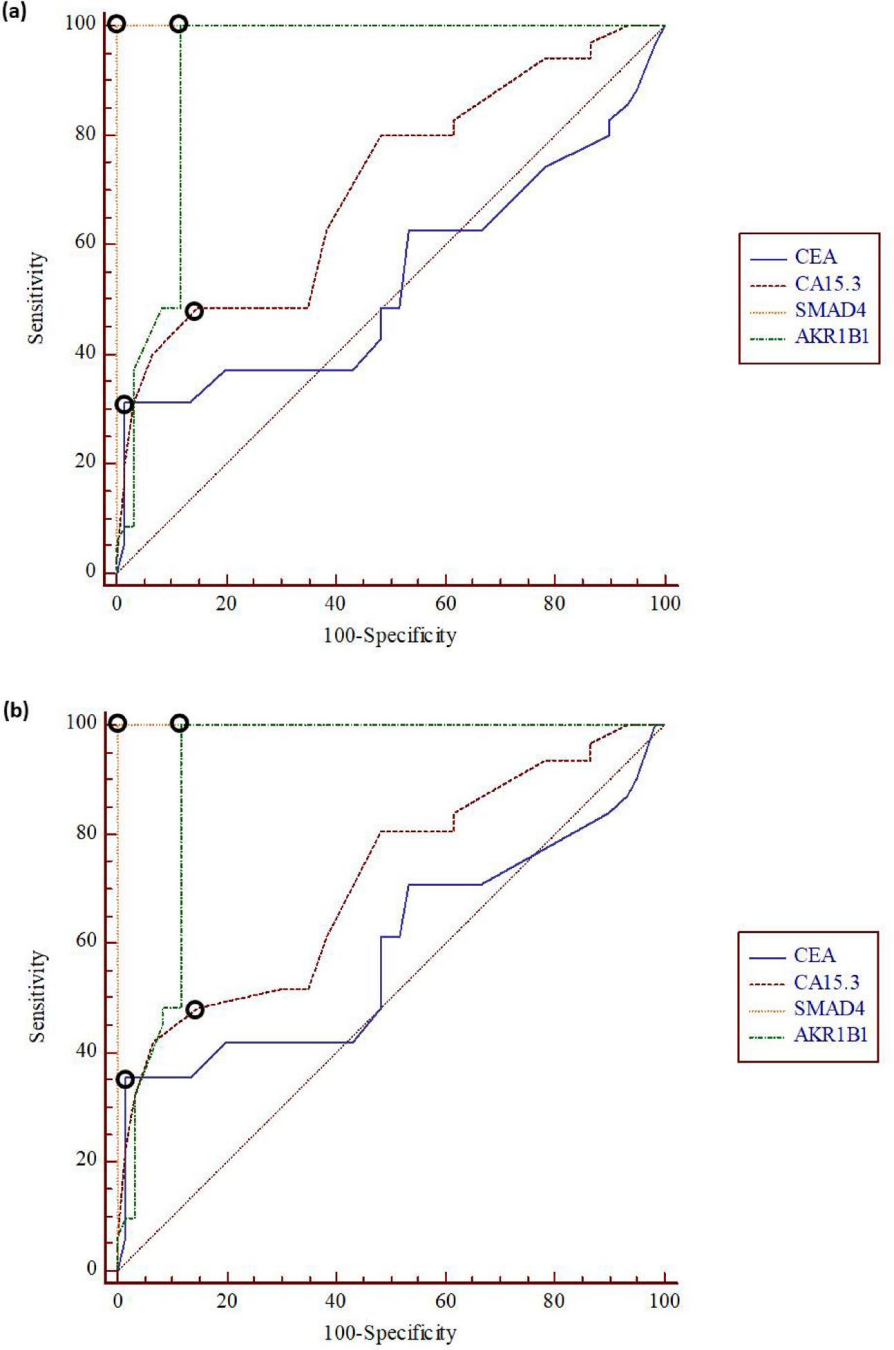
Receiver operating characteristic (ROC) curve for investigated items among **a** early stage and **b** low grade tumors.

The efficacy of tumor markers and methylation status for detecting early-stage and low-grade tumors was assessed using the ROC curve analysis. As illustrated in (Table 4; Fig. 4a, b), for identifying early-stage tumor, the highest AUC was observed for SMAD4 (1), followed by AKR1B1 (0.924), then CA15.3 (0.707), and CEA (0.536) exhibited the lowest AUC. Moreover, SMAD4 and AKR1B1 exhibited superior sensitivity (100%, and 100%, respectively), and specificity (100%, and 88.33%, respectively) over CEA (31.43%, and 98.33%, respectively), and CA 15.3 (48.57%, and 85.00%, respectively) for recognizing patients at the early stage. In low-grade tumors, SMAD4 demonstrated the highest AUC (1), followed by AKR1B1 (0.922), whereas CA15.3 (0.713), and CEA (0.587) exhibited the lowest values. In addition, SMAD4 showed superior diagnostic sensitivity and specificity (100%, and 100%, respectively) compared to CAE (35.48%, and 98.33%, respectively) and CA15.3 (41.94, and 93.33%, respectively) for the recognition of low-grade tumors. Whereas AKR1B1 exhibited superior diagnostic sensitivity (100%) compared to traditional tumor markers.

**Table 4:** Sensitivities and specificities of investigated parameters for detection of early stages and low grades.

Parameters	Early - stage			Low - grade		
	AUC	Sensitivity	Specificity	AUC	Sensitivity	Specificity
CEA	0.536	31.43	98.33	0.587	35.48	98.33
CA15.3	0.707	48.57	85.00	0.713	41.94	93.33
*SMAD4*	1	100.00	100.00	1	100.00	100.00
*AKR1B1*	0.924	100.00	88.33	0.922	100.00	88.33

**Fig. 5:**
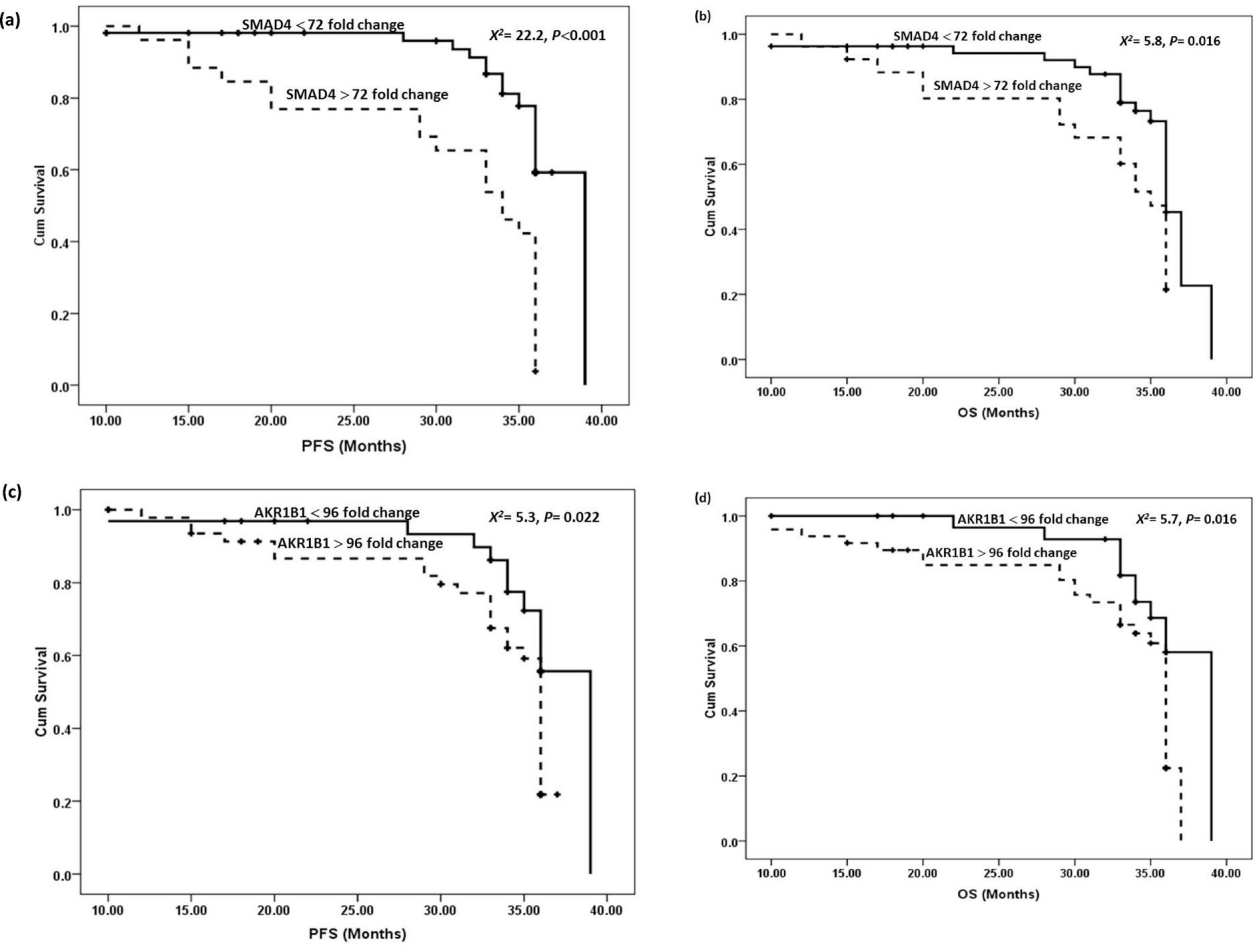
Kaplan–Meyer survival curves. **a** PFS for SMAD4, **b** OS for SMAD4, **c** PFS for AKR1B1and **d** OS for AKR1B1.

### Relation between SMAD4 and AKR1B1 and response to treatment

Table 5 indicates a significant difference in methylation status regarding both items and treatment response, with individuals exhibiting progressive disease demonstrating the highest methylation patterns.

**Table 5:** Mean levels of methylation pattern regarding response to treatment.

Treatment response	SMAD4 (fold change)	AKR1B1 (fold change)
Complete response (CR) *n* = 30	64.3 ± 3.2	74 ± 2.5
Partial response (PR) *n* = 27	73.2 ± 2.5	100 ± 1
Stable disease (SD) *n* = 9	80.5 ± 4.7	109 ± 3.7
Progressive disease (PD) *n* = 14	81 ± 4.3	112 ± 3
Statistics	F = 4.9, *P* = 0.004	F = 61.6, *P*<0.001

### Survival patteren regarding methylation status of investigated genes

Breast cancer patients exhibited heightened methylation levels for the examined genes, correlating with poorer DFS and OS outcomes as plotted in Fig. 5a-d.

## Discussion

Cancer prognosis is crucial in designing the treatment strategies and assessing their effectiveness, underscoring the necessity for a comprehensive understanding^26^. Clinicians need prognostic estimations to differentiate cases with varying molecular types of breast cancer, inform treatment decisions, including individualized therapies for distinct molecular types, manage follow-up, and predict survival time^27^. Previous studies have identified various molecular markers relevant to the prognosis and treatment of breast cancer; however, a consensus on their clinical application is lacking^28,29^.

Conventional clinicopathological prognostic markers, like size of tumor, lymph node metastasis, and histological grade, are inadequate for guiding early-diagnosed breast cancer patients in the context of personalized treatment^28^^,^^30^. Among the biomolecular markers associated with breast cancer, PR, ER, HER2, and the Mib1/Ki-67 proliferation index require invasive investigation^31^^,^^32^. Traditional tumor markers, such as CEA and CA15- 3, remain controversial because of their inadequate specificity and sensitivity, particularly in breast cancer cases at the early stage^33^.

Hence, there is a crucial need to recognize improved predictive and prognostic biomarkers to differentiate patients at high risk for disease progression or relapse. The identification of epigenetic mutations serves as a foundational step in the development of more precise diagnostic and prognostic methods for malignant diseases^4,34^. Consequently, the authors examined serum concentrations of conventional tumor markers (CEA and CA15.3) alongside the methylation patterns of the SMAD4 and AKR1B1 genes. The current results indicate that the mean levels of tumor markers and the methylation status of the examined genes were significantly higher in breast cancer cases versus to benign cases and control individuals, aligning with findings from previous studies^35,36^.

The SMAD4 gene is reported to exhibit dual activity in breast tumorgenesis, functioning either as a suppressor or promoter of cancer progression according to multiple studies^35,37,38^. Nevertheless, all these investigations excluded cases with benign breast lesion and did not address the diagnostic or prognostic effect of SMAD4 methylation in breast cancer. Furthermore, based on our knowledge, there are limited researches addressing the role of epigenetic mutations of SMAD4 in initiation and progression of breast cancer. Other investigations also have revealed a significant role of SMAD4 methylation in other malignancies^39–41^. However, our outcomes were in disagreement with several studies which demonstrated that hypermethylation of promoter area was not observed in colorectal, small intestinal neuroendocrine malignancie, and pancreatic and endometrial cancers^42–44^. Such variation in the SMAD4 methylation across cancers could be explained by the sample size, and the methodology used.

The analysis of AKR1B1 methylation patterns aligns with prior studies that establish a correlation between AKR1B1 gene methylation and the initiation and progression of breast cancer^45,46^. However, these studies did not include benign breast conditions. The current results align with the previous findings of El-Far et al.^47^, which demonstrated that hypermethylation of AKR1B1 is more prevalent in cancerous group than in cases with benign lesions and healthy controls, suggesting a significant correlation with breast cancer incidence (*P* < 0.0001). Furthermore, the diagnostic efficacy of AKR1B1 methylation was notable, exhibiting an AUC of 0.909, which exceeds that of conventional markers such as CA 15 − 3 and CEA.

Our results indicate that hypermethylation of SMAD4 and AKR1B1 is associated with clinicopathological factors, including invasive ductal carcinoma (IDC), advanced stage, and high grades. Hypermethylation of SMAD4 is associated with positive lymph node invasion. The present study indicates that the silencing of SMAD4 and AKR1B1 may be crucial to advancing BC and could function as a novel marker for prognosis of the disease. The findings corroborate those of Swellam et al.^36^ regarding Egyptian breast cancer patients, indicating that elevated SMAD4 methylation status correlates with advanced stages and positive lymph node involvement, thereby suggesting a relationship between hyper-methylation and tumor aggressiveness. For AKR1B1 gene, El-Far et al.^47^ supported our results and noticed that hypermethylated promoter of AKR1B1 gene was associated with late stages, and high histological grades. Luo et al.^1^ showed that promoter hypermethylation of a panel of genes, including AKR1B1, differed significantly in lymph node-positive breast cancer tissues relative to lymph node-negative breast cancer tissues, which is in contradiction to our findings.Our findings indicate a notable difference between the methylation patterns of SMAD4 and AKR1B1 in the breast cancerous group. As far as we are aware, this is the first study to investigate the correlation between SMAD4 and AKR1B1 at the methylation level. This finding can point out the correlation between both among breast cancer patients which need further investigations to elaborate the reason for this correlation.

AKR1B1 is predominantly overexpressed in cancer. This upregulation has been associated with cell cycle mediators, inflammatory mediators, regulatory agents, as a reaction to prostaglandin and reactive oxygen species (ROS) production, as well as signaling pathways and survival proteins like the mammalian target of rapamycin (mTOR) and protein kinase B (Akt)^20^.

The AKT-mTOR intracellular pathway influences cancerous cells proliferation, metabolism, migration, and drug resistance^48^. Previous reports indicate that the inhibition of AKR1B1 can impede the activation of the mTOR cascade via motivating 5′ adenosine monophosphate-activated protein kinase (AMPK), which hinders the mTOR, eIF4E, Raptor,, S6K, and 4E-BP1 phosphorylation, thereby obstructing the development and growth of tumors^20,49^. Previous research highlights the significance of analyzing the inadequate control of TGF-β and its capability for activating various pathways, including phosphoinositide 3-kinases (PI3K), mitogen-activated protein kinases (MAPKs), nuclear factor kappa B (NF-kB), and serine/threonine protein kinases (AKT)^50^. The deregulation of the PI3K/AKT/mammalian target of rapamycin (mTOR) cascade has been related to cancerous diseases^51^. The TGF-β/SMAD4 pathway is extensively regulated by various classical pathways, including the phosphatidylinositol-3 kinase/AKT (PI3K/AKT)^52^. The PI3K/AKT intracellular signaling pathway is commonly associated with cell proliferation and apoptosis inhibition, playing an essential role in the development and progression of multiple malignant diseases^56^. Activation of receptor tyrosine kinases (RTKs) by a ligand stimulates PI3K, converting phosphatidylinositol-4,5-bisphosphate (PIP2) into phosphatidylinositol-3,4,5-triphosphate (PIP3), which acts as a stimulator for the phosphorylation and activation of AKT, which subsequently localizes to the plasma membrane^57^. The canonical signaling pathway of TGF-β/SMAD4 initiates with the binding of the TGF-β ligand to its receptors (TGF-β receptor type II and type I), leading to the SMAD2/3 phosphorylation. After phosphorylation, in conjunction with SMAD4, forms a heterodimeric complex that responsible for nuclear translocation, binds to SBE, and regulates target gene transcription in the presence of transcription factors. The target genes, including tumor suppressor genes, are primarily involved in growth arrest and apoptosis. The PI3K/AKT pathway negatively regulates the TGF-β/SMAD4 pathway by inhibiting the T-βR I phosphorylation, a process promoted by SMAD3 via mTOR. AKT promotes the direct phosphorylation of FOXO, leading to its retention in the cytoplasm, which inhibits its interaction with the promoters of p27 and p21, thus obstructing the cytostatic signals mediated by TGF-β/SMAD4 [Zhao et al., 2018]. TGF-β activation results in the continuous association of the serine/threonine kinase receptor type II with the PI3K subunit (p85). This interaction promotes AKT activation, which in turn inhibits the tuberous sclerosis complex (TSC) and activates mTORC. The activation of mTORC1 facilitates translation of mRNA and rises cell size throughout the epithelial–mesenchymal transition (EMT), migration, and metastatic process. The activation of mTORC2 by TGF-β leads to the activation of AKT^52^.

Considering the efficacy of the examined parameters in identifying early-stage and low-grade tumors, our results showed that SMAD4 methylation patterns, as indicated by the highest AUC (1), exhibited superior sensitivity and specificity compared with CAE and CA15.3 in recognizing early-stage and low- grade tumors. Whereas AKR1B1 demonstrates superior sensitivity compared to CEA and CA15.3. Similar findings have been reported in previous studies, reinforcing our findings. Hai et al^53^. and Hum and Lee^54^ reported that DNA methylation alterations occur early in cancer development, highlighting their potential as biomarkers for early detection. In addition, previous studies stated that SMAD4 promoter hypermethylation, along with decreased SMAD4 protein expression, worsens prognosis in BREAST CANCER. Moreover, evaluating SMAD4 status via biopsy specimens may give appreciated prognostic data, helping in the early detection and treatment approaches^38,55^. Additionally, Chaudhary et al.^55^ indicated that based on SMAD4 promoter hypermethylation, its expression declines from grade 1 to grade 3 BREAST CANCER. This proposes that evaluating SMAD4 methylation status could give precious prognostic data, even in low-grade BREAST CANCER cases. Regarding AKR1B1, in agreement with our outcomes, El-Far et al.^47^ demonstrated that AKR1B1 achieved an AUC of 0.899 for the detection of early-stage, supporting its effectiveness for recognizing malignancies at an early stage.

The epigenetic silencing of tumor-suppressor gene expression affects treatment response and contributes to drug resistance^58^. Consequently, we examined the relationship between the identified patterns and treatment response. Our findings indicated a significant difference in methylation status for both SMAD4 and AKR1B1 in relation to treatment outcomes, with patients exhibiting progressive disease showing the highest levels of methylation.

Regarding SMAD4, two studies in colorectal cancer (CRC) similarly indicated that the inactivation of SMAD4 promoted drug resistance in both in vitro and in vivo contexts^59,60^. Our findings align with existing literature, indicating that diminished SMAD4 activity is associated with carcinogenesis, chemoresistance, immune cell infiltration, and unfavorable prognosis across multiple cancer types^61–63^. A meta-analysis indicates that reduced SMAD4 expression is linked to increased drug resistance, recurrence, as well as metastasis across various malignancies, including BREAST CANCER. Xu et al.^64^ and Li et al.^65^ recently indicated that the downregulation of SMAD4 expression is linked to worse prognosis and causes endocrine treatment resistance in ER-positive BREAST CANCER, adversely impacting treatment outcomes. Zhang et al.^66^ concluded that two histone methyltransferases contribute to drug resistance in non-small-cell lung cancer (NSCLC) via facilitating the silencing of the SMAD4 gene. Other studies have indicated that the inactivation of SMAD4 does not significantly correlate with the sensitization of pancreatic cancer cells to cisplatin or their drug resistance^67,68^. Discrepancies between our outcomes and those of other studies may arise from complex resistance mechanisms, including genetic alterations and various signaling pathways, which could contribute to treatment failure or drug resistance^69^. Prior studies indicated that cross-resistance to various medications may also contribute to resistance mechanisms^70,71^. Our findings regarding AKR1B1 align with previous and recent studies on prostate cancer patients, which concluded that AKR1B1 can effectively distinguish between responders and non-responders to treatment, achieving AUCs of 0.931^72^.

Our analysis of methylation biomarkers indicated that higher methylation levels for the examined items were associated with poorer PFS and OS.

The assessment of SMAD4 protein levels in breast ductal carcinoma biopsy samples may provide further prognostic information. As demonstrated by Liu et al.^73^ cases with significantly lower SMAD4 expression levels exhibited more poorly differentiated tumors, a higher recurrence rate, and shorter OS. A meta-analysis by Xu et al.^64^ revealed that a decline in SMAD4 expression correlated strongly with worse prognosis for OS or RFS in patients treated with chemotherapeutic agents. For AKR1B1, Dillinger et al.^72^ reported significant associations between higher methylation levels and reduced radiographic progression-free survival (rPFS) for several markers, including the AKR1B1 gene, using the Kaplan-Meier method.

## Conclusion

The current study highlights the clinical relevance of SMAD4 and AKR1B1 methylation in breast cancer as significant prognostic biomarkers, suggesting their role in predicting treatment response and survival outcomes.

### Limitations of the study

The present study indicates a correlation between SMAD4 and AKR1B1 gene methylation in breast cancer; however, certain limitations are noted. Specifically, the signaling pathways that underscore this correlation were not examined, indicating that future research is necessary to elucidate them. Moreover, gene expression or protein levels related to the aforementioned items in BREAST CANCER samples were not analyzed, which could have elucidated the relationship between SMAD4 and AKR1B1 gene methylation and their respective gene expression or protein levels. Consequently, future research is underway to address this gap. Furthermore, it is recommended that a combined analysis of SMAD4 and AKR1B1 methylation could be conducted in future studies for the diagnosis and prognosis-predicting of BREAST CANCER patients.

## Data Availability

The datasets produced and/or analyzed throughout this current investigation are not available to the public but can be taken from the corresponding author upon a reasonable request.

## Abbreviations

BC: Breast cancer
SMAD4: Small mothers against decapentaplegic 4
AKR1B1: Aldo-keto reductase family 1 member B1
ROC: Receiver operating characteristic
OS: Overall survival
PFS: Progression free survival
AUC: Area under the curve
CA 15 − 3: Cancer antigen 15 − 3
CEA: Carcinoembryonic antigen
TGF-β: Transforming growth factor-β
EMT: Epithelial mesenchymal transition
BLBREAST CANCER: Base like- breast cancer
IDC: Invasive duct carcinoma
DCI: Duct carcinoma in situ
ELISA: Enzyme linked immunesorbent assay
qPCR: Quantitative polymerase chain reaction
LN: Lymph node
ROS: Reactive oxygen species
mTOR: Mammalian target of rapamycin
MAPKs: Mitogen-activated protein kinases
NF-kB: Nuclear factor kappa B
PI3K: Phosphoinositide- 3-kinases
PIP2: Phosphatidylinositol-4,5-bisphosphate
PIP3: Phosphatidylinositol-3,4,5-triphosphate
